# Type 17-specific immune pathways are active in early spondyloarthritis

**DOI:** 10.1136/rmdopen-2023-003328

**Published:** 2023-12-20

**Authors:** Catherine D Hughes, Sarah E Ryan, Kathryn J A Steel, Michelle D van den Beukel, L A Trouw, Karin A J van Schie, René E M Toes, Bina Menon, Bruce W Kirkham, Leonie S Taams

**Affiliations:** 1 Centre for Inflammation Biology & Cancer Immunology, King's College London, London, UK; 2 Immunology, Leiden University Medical Center, Leiden, Netherlands; 3 Department of Rheumatology, Leiden University Medical Center, Leiden, Netherlands; 4 Department of Rheumatology, Guy's and St Thomas' Hospitals NHS Trust, London, UK

**Keywords:** Arthritis, Psoriatic, Arthritis, Rheumatoid, Cytokines, Synovial fluid, T-Lymphocyte subsets

## Abstract

**Objective:**

Undifferentiated, early inflammatory arthritis (EIA) can differentiate into seropositive or seronegative rheumatoid arthritis (RA), peripheral spondyloarthritis (SpA) or remain as seronegative undifferentiated inflammatory arthritis (UIA). Little is known about immune pathways active in the early stages of SpA and seronegative UIA, in contrast to detailed knowledge of seropositive RA. The aim of this study was to examine if specific immune pathways were active in synovial CD4+ and CD8+ T cells in EIA.

**Methods:**

Synovial fluid (SF) samples from 30 patients with EIA were analysed for expression of IL-17A, IFNγ and TNFα in CD8+ or CD4+ T cells. Final clinical diagnoses were made at least 12 months after sample collection, by two independent clinicians blind to the study data.

**Results:**

Flow cytometry analysis of all EIA samples indicated considerable variation in synovial IL-17A+CD8+ T cells (Tc17) cell frequencies between patients. The group with a final diagnosis of SpA (psoriatic arthritis or peripheral SpA, n=14) showed a significant enrichment in the percentage of synovial Tc17 cells compared with the group later diagnosed with seronegative UIA (n=10). The small number of patients later diagnosed with seropositive RA (n=6) patients had few Tc17 cells, similar to our previous findings in established disease. In contrast, RA SF contained a significantly higher percentage of CD8+IFNγ+ T cells compared with SpA or seronegative UIA.

**Conclusion:**

These results suggest that adaptive T cell cytokine pathways differ not only between RA and SpA but also seronegative UIA early in the disease process, with a particular activation of Tc17 pathways in early SpA.

**WHAT IS ALREADY KNOWN ON THIS TOPIC**

- Many studies have reported on the presence of pro-inflammatory cytokine-expressing T cell subsets in the joints of patients with established rheumatoid arthritis (RA) and spondyloarthritis (SpA). Much less is known about the immune pathways that are active in early inflammatory arthritis (EIA), particularly in early SpA and seronegative undifferentiated inflammatory arthritis (UIA). We aimed to examine if specific immune pathways are active in synovial CD4+ and CD8+ T cells from patients with EIA.

**WHAT THIS STUDY ADDS**

- Our results suggest that adaptive T cell cytokine pathways differ already early in the disease process between patients who go on to be diagnosed with seropositive RA versus those later diagnosed with seronegative SpA or UIA. We report evidence for a particular activation of type 17 pathways in CD8+T cells in early SpA.

**HOW THIS STUDY MIGHT AFFECT RESEARCH, PRACTICE OR POLICY**

- These data add to the increasing evidence that the immunopathology of seropositive RA and seronegative SpA and UIA is different.

## Introduction

Early inflammatory arthritis (EIA) can be described as a duration of inflammatory features of greater than 6 weeks but less than 1–2 years.^1–3^ Patients in this category are classically defined as having seronegative undifferentiated arthritis if they do not satisfy the criteria for diagnosis of rheumatoid arthritis (RA), psoriatic arthritis (PsA) or axial and peripheral spondyloarthritis (SpA).

It is increasingly recognised that there are distinct differences in the clinical manifestations, genetics, serological and cellular features between established RA and SpA/PsA,^4–6^ with much less research into undifferentiated arthritis or seronegative RA. In contrast to RA, patients with SpA/PsA can have significant extra-articular disease associations (psoriasis, inflammatory bowel disease, uveitis).^7^ Furthermore, PsA is associated with increased body mass index in many patients with concomitant metabolic syndrome and cardiovascular morbidity.^8^ There are strong associations between seropositive (ACPA+) RA and cigarette smoking and *HLADRB1* shared epitope alleles.^3 9–12^ These associations are not observed in seronegative RA. Instead, it has been suggested that *HLADRB3* and interferon regulatory factor 5 (*IRF5*) may predispose to seronegative RA.^13–15^ It is also unclear as to whether seronegative RA is one or several disease entities.^3^ Finally, we recently reported quantitative differences in the composition of synovial tissue-resident memory T cells between patients with established RA versus PsA.^5^

Key cytokine pathways with broad unified activities have been identified, with the type 1 pathway indicated by interferon-gamma (IFNγ), and the type 17 pathway indicated by IL-17A.^16^ These two pathways are known to be expressed in the joints of patients with inflammatory arthritis. IFNγ is a potent activator of myeloid cells and of MHC class I/II expression, highlighting its proinflammatory role, although it should be noted that certain aspects of its pathogenic action in inflammatory arthritis remain incompletely elucidated.^17 18^ IL-17A is a proinflammatory cytokine with broad-ranging effects on angiogenesis, neutrophil infiltration and stromal cell activation.^19–21^ In the context of inflammatory arthritis, IL-17A can synergise with TNFα to promote osteoclast activation^22^ and induce proinflammatory cytokine and chemokine production from fibroblasts and synoviocytes.^23–27^ Paradoxically, and relevant to SpA, IL-17A has also been shown to promote new bone formation in HLA-B27/hβ(2) m-transgenic rats.^28^ Our previous work demonstrated that IL-17A+CD8+ T cells with a tissue-resident profile are enriched in the synovial fluid (SF) of patients with established PsA/SpA, but not in patients with RA suggesting that IL-17 expression is an important differentiating pathway between these two diseases.^5 29 30^

To enhance our understanding of the lymphoid cytokine pathways that may operate early on in the disease process of inflammatory arthritis, we investigated T cell expression of IL-17, IFNγ and TNFα in the inflamed joints of patients with EIA.

## Materials and methods

### Clinical diagnosis

We enrolled patients with early inflammatory arthritis which we defined as symptoms of arthritis of no more than 12 months duration, with an initial clinical diagnosis of an inflammatory arthritis including RA, SpA—which included PsA, enteric-related IA and reactive arthritis—and seronegative undifferentiated inflammatory arthritis (UIA). Patient samples were collected between Feb 2014 and May 2021 (online supplemental table 1). Final clinical diagnoses were independently applied by two investigators (BWK and BM) who were blind to each other’s diagnosis and research laboratory data, using clinical, imaging and laboratory data including rheumatoid factor, ACPA and ANA autoantibodies, from patient electronic records. Diagnoses were compared and two patients with slightly different diagnoses were discussed to complete a final consensus diagnosis. The changes in diagnosis from the time SF samples were taken to final diagnoses were: four patients initially diagnosed as reactive arthritis, with a weak history of a preceding infection arthritis, developing into an undifferentiated inflammatory arthritis (UIA) pattern of disease, one patient diagnosed with UIA who subsequently had a preceding infection confirmed with a final diagnosis of reactive arthritis, one patient diagnosed as UIA and another with reactive arthritis who subsequently developed a PsA pattern of disease and one patient initially diagnosed with PsA who did not have psoriasis with a final diagnosis of UIA.

10.1136/rmdopen-2023-003328.supp1 Supplementary data

### Samples and cell isolation

SF samples from patients with EIA were collected following written informed consent by the Rheumatology Department at Guy’s Hospital (REC reference 06/Q0705/20 and 17/LO/1940). Patient demographic and clinical information is shown in table 1. SF mononuclear cells (SFMCs) were isolated by density gradient centrifugation using Lymphoprep (Alere Technologies) and cryopreserved in liquid nitrogen until use.

**Table 1:** Patient demographic and clinical information

n	SpA	Seronegative UIA	Seropositive RA
	14	10	6
Disease duration months, mean (range)	5.13 (0.5–12)	3.75 (2–7)	5.33 (1–11)
Age, mean (range)	30 (23–52)	48 (21–79)	52 (25–84)
Female, n (%)	4 (29)	6 (60)	5 (83)
No treatment, n (%)	4 (29)	2 (20)	5 (83)
NSAID*, n (%)	8 (57)	1 (10)	1 (17)
DMARD†, n (%)	1 (7)	6 (60)	0
Biologic‡, n (%)	1 (7)	0	0
Steroid§, n (%)	1 (7)	5 (50)	1 (17)
ACPA+, n (%)¶	0	0	3/4 (75%)
Anti-CarP+, n (%)¶	0	1/5 (20%)	2/4 (50%)

Demographic and clinical information for patients included in this study.

*Naproxen, etoricoxib and ibuprofen.

†Methotrexate and sulfasalazine.

‡Guzelkumab.

§Prednisolone.

¶Serum available for ACPA/anti-CarP testing SpA n=7, seronegative UIA n=5, seropositive RA n=4.

ACPA+, anti-citrullinated peptide antibody positive; Anti-CarP, anti-carbamylated peptide antibody positive; DMARD, disease-modifying antirheumatic drug; NSAID, non-steroidal anti-inflammatory drug; SpA, spondyloarthritis; UIA, undifferentiated inflammatory arthritis.

### Flow cytometric analysis

For intracellular cytokine staining, SFMC were thawed, rested for 1 hour and then stimulated in culture medium (RPMI 1640 with 1% Pen/Strep/Glutamine and 10% fetal calf serum (FCS)) with phorbol myristate acetate (PMA) (50 ng/mL) and ionomycin (750 ng/mL, both Sigma-Aldrich) for 3 hours at 37°C in the presence of GolgiStop (monensin, according to manufacturer’s recommendation, BD Biosciences). Cells were washed and labelled with fixable viability dye (LIVE/DEAD eFluor780, eBioscience), alongside extracellular staining with mouse-anti-human antibodies (see table 2 for details), for 20 min at 4°C. Cells were fixed with 2% paraformaldehyde for 15 min at 4°C, then permeabilised using 0.5% saponin (Sigma-Aldrich) and stained for 30 min at 4°C with the mouse-anti-human antibodies. For fluorescence minus (FM) control staining, small sample aliquots were combined and stained as above but without addition of anti-cytokine mAbs. Samples were acquired using either a FACS CantoII or LSRFortessa (BD Biosciences). Flow cytometry data were analysed using FlowJo software (V.10.8.1, TreeStar). The vast majority of samples were stained and acquired between January 2018 and December 2019. Once all samples were acquired, an analysis pipeline was set up and applied to ensure that all samples were gated and analysed in an identical manner (analysis performed between July 2020 and July 2021). For this, CD8+ and CD4+ T cells were gated followed by FM control gating, which was set separately for each individual sample and cytokine to aid determination of cytokine-expressing cell populations (online supplemental figure 1).

**Table 2:** Antibodies used for sample staining

Epitope	Fluorochrome	Clone	Company
CD3	PE-Cy7	UCHT1	Biolegend
CD3	BUV737	UCHT1	Biolegend
CD4*	PerCP-Cy5.5	SK3	Biolegend
CD4*	BV785	RPA.T4	Biolegend
CD8	Pacific Blue	RPA-T8	Biolegend
CD8	BUV395	RPA-T8	Biolegend
CD14	APC-Cy7	REA559	Miltenyi
CD19	APC-Cy7	HIB19	Biolegend
IFNγ*	FITC	B27	Biolegend
IFNγ*	PerCP-Cy5.5	4S.B3	Biolegend
IL-17A*	PE	BL168	Biolegend
TNFα*	APC	Mab11	Biolegend
TNFα*	FITC	Mab11	Biolegend

Antibodies used for sample staining.

*Indicates these antibodies were added intracellularly.

### Autoantibody analysis

Almost all patients (90%) had routine ACPA testing at our centre at the time of SF sampling, using the Elia test (Thermo-Fisher, with a Phadia250 instrument). To extend our characterisation of seronegative undifferentiated arthritis patients, we also determined ACPA antibodies and anti-CarP antibodies, in patients with saved serum. These ACPA IgG antibody levels were determined using an in-house ELISA, essentially as described before.^31^ In short, streptavidin coated plates were incubated with biotinylated cyclic citrullinated peptide 4 or the arginine control peptide. In between each sequential step, plates were washed five times with PBS/0.05%Tween. Serum samples, diluted 1/50 in PBS/1% BSA/0.05% Tween, were added to the plate and incubated for 1 hour at 37°C. Next, ACPA IgG were detected as described above. ACPA IgG binding was quantified relative to a standard curve and expressed in arbitrary units per mL. A cut-off based on healthy controls was determined as described above. Patient samples were considered positive if the ACPA level was higher than the cut-off, as well as >2 times higher than the control peptide.

Anti-CarP IgG antibody levels were detected using an in-house ELISA as described previously.^32^ In brief, a Nunc Maxisorp plate (Thermofisher) was coated with carbamylated FCS or non-modified FCS. In between each sequential step, plates were washed three times using PBS/0.05%Tween (Sigma). Plates were blocked for 6 hours at 4°C and were then incubated with 1/50 diluted serum overnight at 4°C. After incubation, IgG levels were detected using Rabbit-anti-Human IgG-HRP (Dako). Plates were developed using 2,2’-azino-bis(3-ethylbenzothiazoline-6-sulfonic acid) (ABTS)/0.015% H2O2 (both from Merck) and absorbance was measured at 415 nm. Antibody binding was quantified relative to a standard line and expressed in arbitrary units per mL. Next, antibody binding to the control proteins was subtracted from the antibody binding to carbamylated protein. The cut-off for positivity was set as the mean arbitrary units plus two times the SD of 100 healthy controls.

### Statistical analysis

Graphs were constructed with GraphPad Prism V.9.5.1. Statistical analysis was performed using the Kruskal-Wallis test followed by Dunn’s multiple comparisons test. P values were considered significant if p<0.05.

## Results

We recruited 45 patients with early inflammatory arthritis with symptoms of no more than 12 months, whose clinical management required SF aspiration from a knee joint. Five patients declined to have SF samples tested, nine samples did not yield sufficient SFMC to analyse and one patient remained with an inconclusive diagnosis. Of the 30 patients analysed, 14 had a final diagnosis of SpA (7 PsA, 2 enteropathic SpA, 5 reactive arthritis), 10 of seronegative undifferentiated inflammatory arthritis (UIA) and 6 of seropositive RA. Serum for detailed autoantibody profiling was available for 16 out of 30 patients. Patient demographic, serology and clinical parameters at the time of SF collection in the 30 tested samples are shown in table 1. The final diagnosis was made at least 12 months (range 12–48 months) after symptom onset independently by two clinicians blinded to the research laboratory data. Final clinical diagnoses were linked to the laboratory data after completion of the flow cytometry acquisition and analysis.

Recent findings suggest some seronegative undifferentiated inflammatory arthritis patients are positive for antibodies such as anti-carbamylated proteins (anti-CarP), which are not yet routinely tested in the clinic.^33^ In addition to our routine laboratory ACPA testing, we assessed serum levels of ACPA and anti-CarP antibodies in 16 of the 30 patients, using in-house assays. Of the tested samples, the sera from patients later diagnosed with SpA were all ACPA and anti-CarP negative (n=7) and those from patients later diagnosed with seronegative UIA (n=5) were all negative for ACPA with one patient testing positive for anti-CarP only. Of the patients later diagnosed with seropositive RA, three and two out of four serum samples tested positive for ACPA and anti-CarP, respectively.

To investigate the presence of specific cytokine-expressing CD8+ or CD4+ T lymphocytes, SFMC were thawed, rested and stimulated for 3 hours with PMA and ionomycin in the presence of GolgiStop before staining for the appropriate markers.

**Figure 1:**
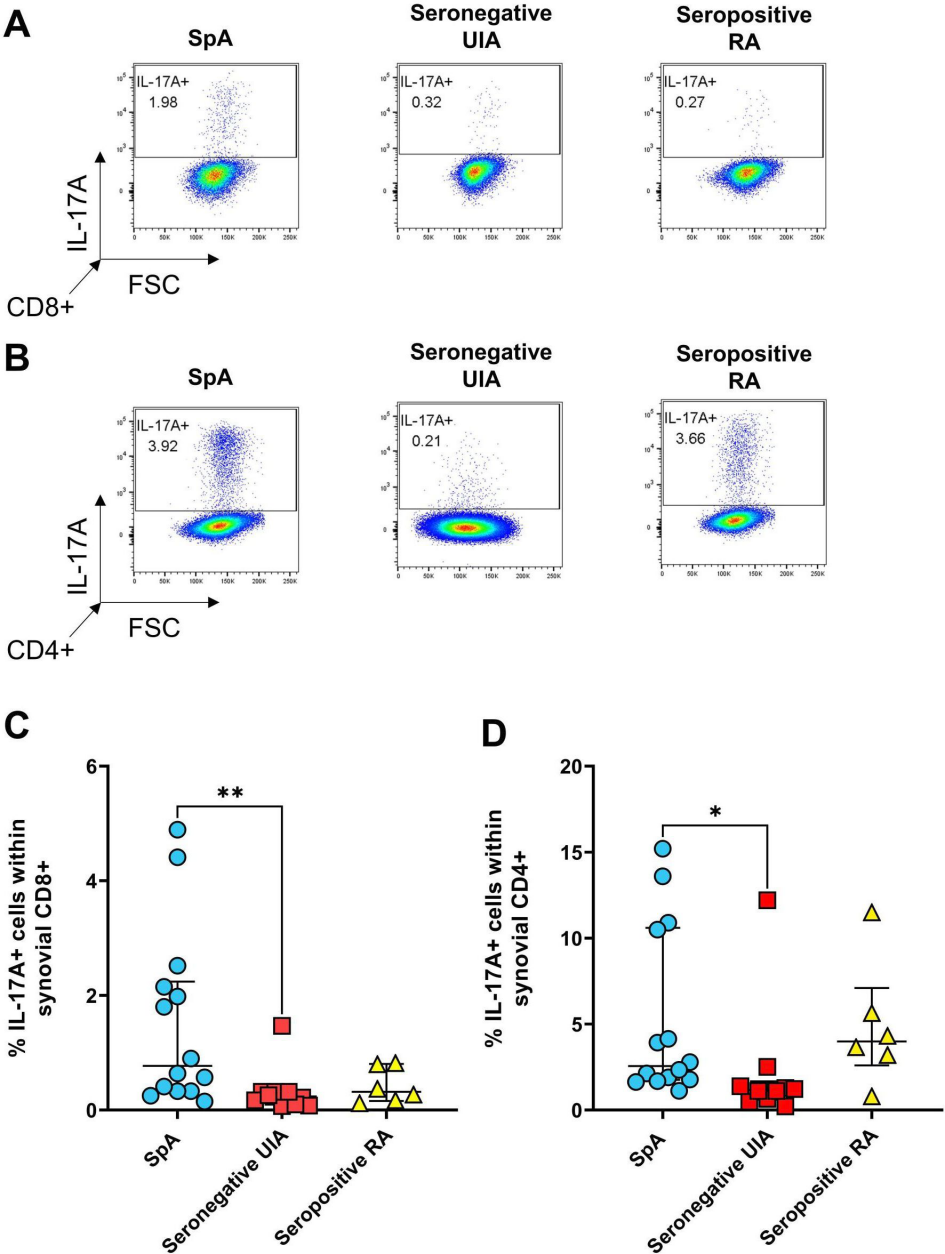
Presence of IL-17A+ CD8+ or CD4+ T-cells in the synovial fluid of patients with EIA. SFMC was stimulated for 3 hours with PMA and ionomycin in the presence of GolgiStop followed by staining for the presence of IL-17A producing T cells. Representative dot plots (A, B) and cumulative data (C, D) showing the presence of IL-17A+ cells within CD8+ (A, C) and CD4+ (B, D) T cells from the synovial fluid of patients with EIA who were subsequently diagnosed as having SpA (n=14, blue circles), seronegative UIA (n=10, red squares) or seropositive RA (n=6, yellow triangles). Data analysed using the Kruskal-Wallis test. EIA, early inflammatory arthritis; PMA, phorbol myristate acetate; RA, rheumatoid arthritis; SFMC, synovial fluid mononuclear cell; SpA, spondyloarthritis; UIA, undifferentiated inflammatory arthritis. * p < 0.05; ** p < 0.01.

Overall, the percentage of IL-17A+CD8+ T cells was significantly enriched (p=0.0054) in the SpA group compared with the group later diagnosed with seronegative UIA (figure 1A and C). A similar pattern was observed for synovial IL-17A+CD4+ T cells (p=0.0253) (figure 1B and D). The small group of patients later diagnosed with seropositive RA (n=6) all had low levels of IL-17A+CD8+ T cells, while the percentage of IL-17A+CD4+ T cells was similar to that in SpA.

Online supplemental figure 2 shows the individual datapoints of the synovial Tc17 or Th17 cell frequencies in the three patients subgroups in relation to age, medication, gender or SpA subgroup. Given the heterogeneity and relatively small sample size in each group, statistical analysis was not possible, but no immediate trends were discernible. We also analysed whether there was any relation between %Tc17 cells and time since first symptom onset or C reactive protein level, but no statistical significance was found (online supplemental figure 3). A negative correlation was found for %Th17 cells and time since symptom onset. Finally, we analysed whether there was a correlation between Tc17 and Th17 cells in the EIA SF samples and found a significant correlation when we compared all samples (n=30), but not when we only included the SpA subset (n=14) which may be due to the lower n-number (online supplemental figure 4).

**Figure 2:**
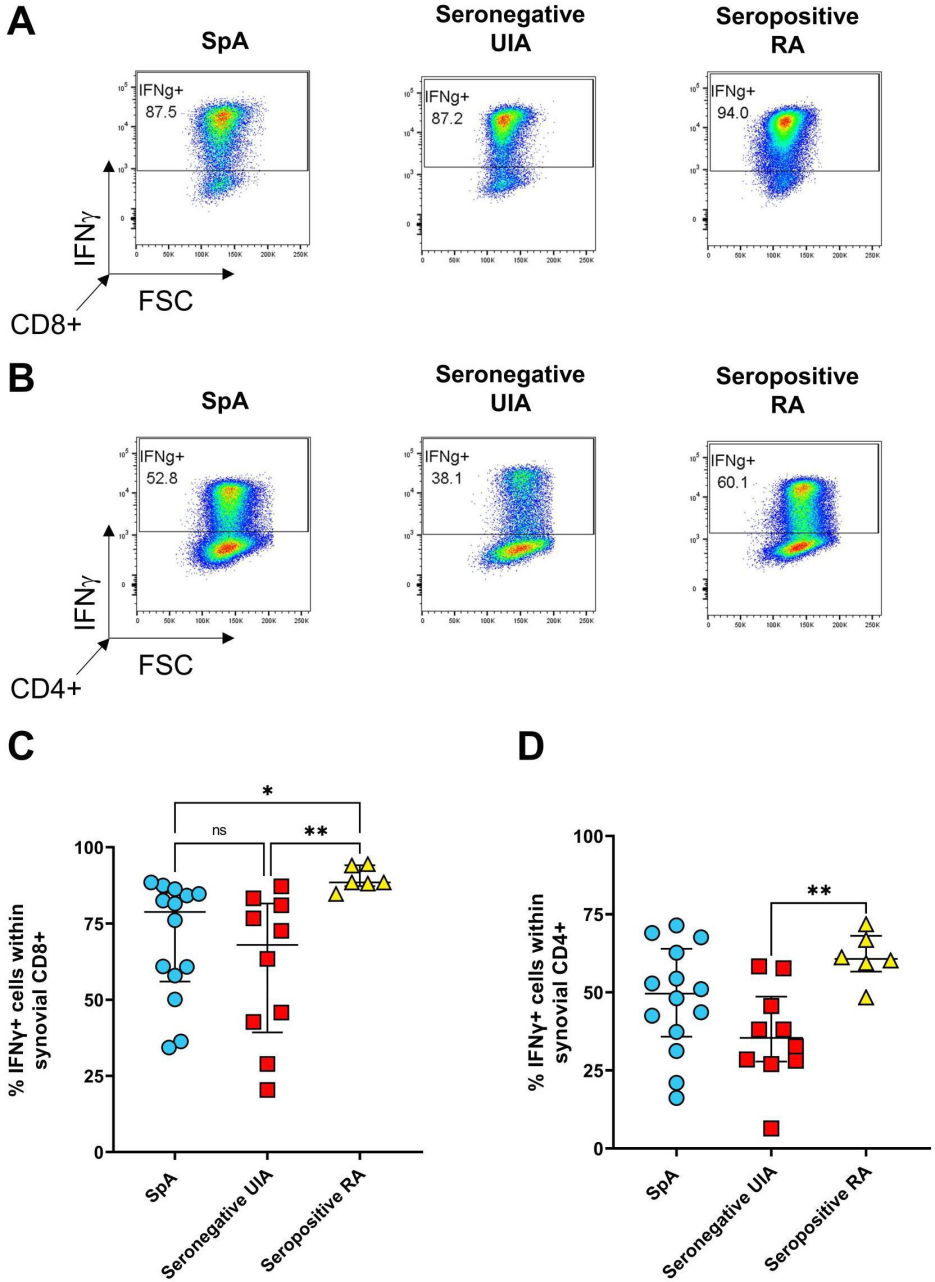
Presence of IFNγ+ CD8+ or CD4+ T-cells in the synovial fluid of patients with EIA. SFMC was stimulated for 3 hours with PMA and ionomycin in the presence of GolgiStop followed by staining for the presence of IFNγ producing T cells. Representative dot plots (A, B) and cumulative data (C, D) showing the presence of IFNγ+ cells within CD8+ (A, C) and CD4+ (B, D) T cells from the synovial fluid of patients with EIA who were subsequently diagnosed as having SpA (n=14, blue circles), seronegative UIA (n=10, red squares) or seropositive RA (n=6, yellow triangles). Data analysed using the Kruskal-Wallis test. EIA, early inflammatory arthritis; PMA, phorbol myristate acetate; RA, rheumatoid arthritis; SFMC, synovial fluid mononuclear cell; SpA, spondyloarthritis. * p < 0.05; ** p < 0.01.

EIA patients later diagnosed with seropositive RA showed a significantly higher percentage of synovial IFNγ+CD8+ T cells compared with both the SpA (p=0.0138) and the seronegative UIA (p=0.0016) groups, and in IFNγ+CD4+ T cells compared with seronegative UIA (p=0.0089) (figure 2).

**Figure 3:**
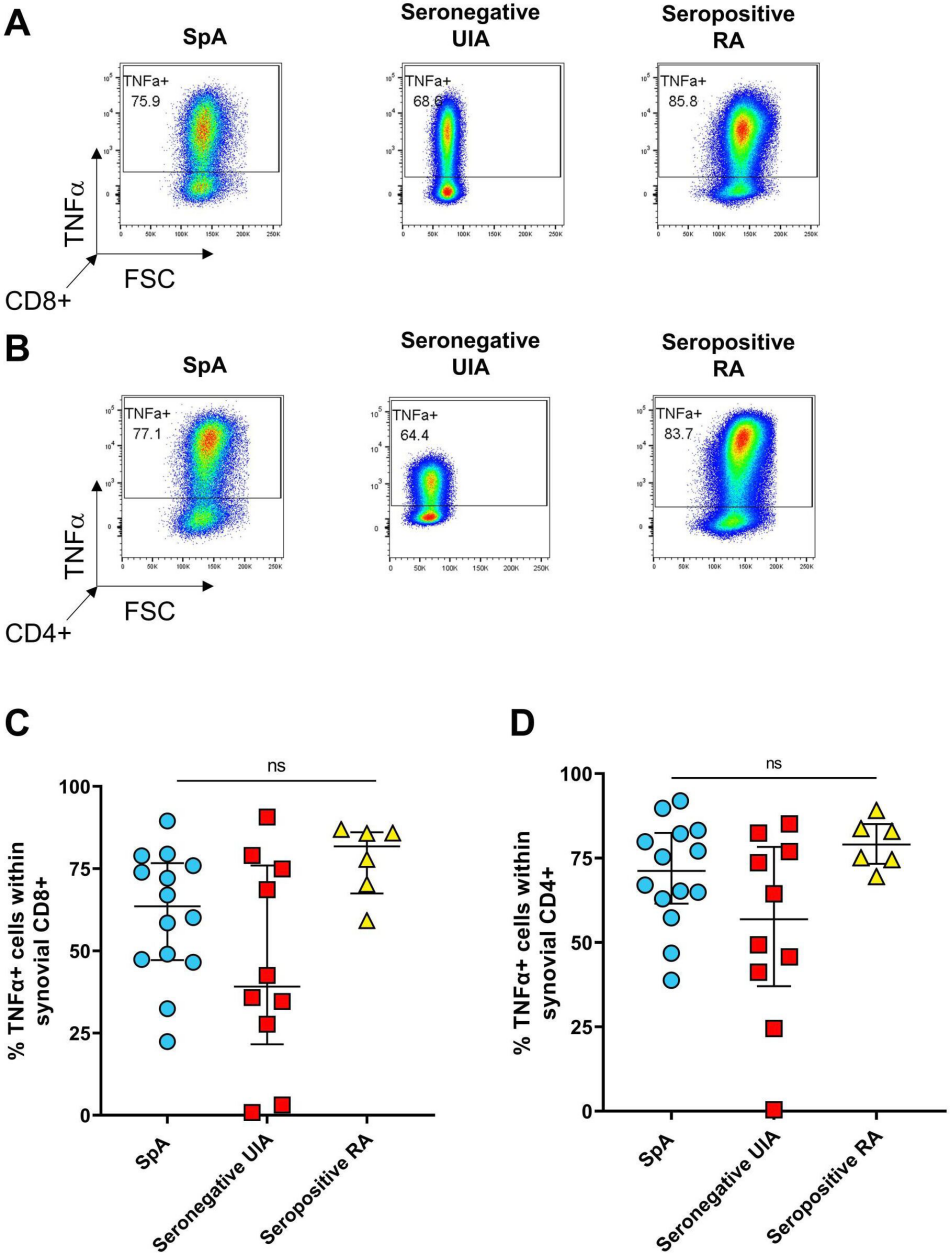
Presence of TNFα+ CD8+ or CD4+ T-cells in the synovial fluid of patients with EIA. SFMC was stimulated for 3 hours with PMA and ionomycin in the presence of GolgiStop followed by staining for the presence of TNFα producing T cells. Representative dot plots (A, B) and cumulative data (C, D) showing the presence of TNFα+ cells within CD8+ (A, C) and CD4+ (B, D) T cells from the synovial fluid of patients with EIA who were subsequently diagnosed as having SpA (n=14, blue circles), seronegative UIA (n=10, red squares) or seropositive RA (n=6, yellow triangles). Data are analysed using the Kruskal-Wallis test. EIA, early inflammatory arthritis; PMA, phorbol myristate acetate; RA, rheumatoid arthritis; SFMC, synovial fluid mononuclear cell; SpA, spondyloarthritis; UIA, undifferentiated inflammatory arthritis.

The percentages of synovial TNFα+CD8+ or CD4+ T cells were not different between EIA patients later diagnosed with SpA, seronegative UIA or seropositive RA (figure 3).

## Discussion

At present, our understanding of the immunological mechanisms driving the early phases of SpA and seronegative UIA is incomplete. Recent evidence indicates that more patients are now presenting with seronegative UIA patterns, which could relate to reduced smoking and increased population adiposity.^34^ A deeper understanding of the underlying immunopathology of diverse forms of inflammatory arthritis is therefore important. In this report, we focused on two key functional immune pathways, namely type 1, characterised by IFNγ expression and type 17 where IL-17A is the signature cytokine, and investigated if these pathways were present early in the disease process of different types of EIA.

Our data indicate that the frequency of SF IL-17A+CD8+ (Tc17) T cells in patients with early SpA, including both PsA and peripheral SpA, is significantly increased compared with the seronegative UIA group and numerically increased compared with the smaller seropositive RA group. We have previously demonstrated significantly elevated synovial Tc17 frequencies in established SpA compared with RA^5 29 30^ but similar enrichment of Th17 cell frequencies,^29 30^ suggesting the patterns we show here in EIA persist over time, despite most patients in the established arthritis group receiving DMARD and biological therapy.

In contrast, we show a significantly higher percentage of type 1 CD8+IFNγ+ SF T cells from patients with early seropositive RA compared with both the seronegative UIA and SpA groups, and in CD4+IFNγ+ SF T cells in early RA versus seronegative UIA. Our findings of elevated CD8+ and CD4+ IFNγ+ SF cells in EIA patients eventually diagnosed with RA support previous reports of an increased type 1/IFNγ signature in seropositive RA.^35–37^ Furthermore, two recent studies reported the presence of clonally expanded cytotoxic CD8+ and CD4+ T cells in the blood and joints of ACPA+RA patients.^38 39^ The cytotoxic CD8+T cells were activated by citrullinated antigens in an HLA class I-dependent manner leading to expression of pro-inflammatory and cytolytic mediators (IFNγ and/or GZMB)^38^ while the cytotoxic CD4+T cells were characterised by a GZMB+PRF1+, Hobit+, NKG7^high^ and GPR56+profile.^39^ Our study focused on type 1 and type 17 cytokine-expressing T cells and did not investigate cytotoxic cell subsets.

Our study is one of few to analyse synovial immunopathology in a broad group of patients with early disease. Previous research in EIA often compared outcomes and pathology in patients with seropositive or seronegative RA, excluding PsA or peripheral SpA.^40 41^

Importantly, our data show significant differences in early disease between the well-studied group of seropositive RA, in contrast to the less well studied PsA/SpA and the seronegative UIA groups. The latter group has been difficult to study as there are no clear classification criteria but is important as these patients are increasingly common in EIA services.^34^ We show that the seronegative UIA group is characterised by lower frequencies of IL-17A and IFNγ-expressing CD8+ and CD4+ T cells. Levels of TNFα-expressing cells are not statistically different across all groups but again in the seronegative UIA group appeared at the lower range of the other two groups.

Do these differences in key immune pathways that we and others have shown in early and established IA, have clinical implications? The clearest clinical relationship is the well-demonstrated response of SpA to IL-17A inhibition therapy in contrast to a much less profound improvement in other types of inflammatory arthritis.^42 43^ Our finding that the Tc17 pathway is active in very early SpA may in part help explain these differences in response to IL-17 inhibition.^44^ Conversely, most arthritis types respond to inhibition of TNF,^45^ which was expressed at similar levels in all the arthritis types tested here.

A limitation of our study is that it contained relatively small numbers, particularly in the seropositive RA group, which makes some comparisons difficult. Furthermore, we did not obtain information on the HLA-B27 status of our patients, which could have helped in the diagnosis of SpA patients. Finally, in SpA it is also recognised that many innate lymphoid cells, such as NK, gamma delta, ILC and MAIT cells express features of the type 17 response, whereas we focused on conventional adaptive T cells. This might underestimate the breadth of the type 17 response in SpA.

## Conclusions

In conclusion, our data show that key type 1 and Tc17 immune activation pathways, that were previously demonstrated to be present in established seropositive RA and peripheral SpA/PsA respectively, are present in these types of inflammatory arthritis at an early stage of the disease process. These findings add to the growing evidence that there are distinct differences in the immunopathology underlying SpA, seronegative UIA and seropositive RA.

## Data Availability

All data relevant to the study are included in the article or uploaded as online supplemental information.

## References

[1] Scott DL. Early rheumatoid arthritis. Br Med Bull 2007;81–82:97–114. 10.1093/bmb/ldm011 17540693

[2] Smolen JS. Undifferentiated early inflammatory arthritis in adults Waltham, MA. 2018.

[3] de Rooy DPC, van der Linden MPM, Knevel R, et al. Predicting arthritis outcomes--what can be learned from the Leiden early arthritis clinic Rheumatology (Oxford) 2011;50:93–100. 10.1093/rheumatology/keq230 20639266

[4] Veale DJ, Fearon U. What makes Psoriatic and rheumatoid arthritis so different RMD Open 2015;1:e000025. 10.1136/rmdopen-2014-000025 26509055 PMC4613157

[5] Povoleri GAM, Durham LE, Gray EH, et al. Psoriatic and rheumatoid arthritis joints differ in the composition of Cd8+ tissue-resident memory T cell Subsets. Cell Rep 2023;42:112514. 10.1016/j.celrep.2023.11251437195862

[6] Floudas A, Smith CM, Tynan O, et al. Distinct Stromal and immune cell interactions shape the pathogenesis of rheumatoid and Psoriatic arthritis. Ann Rheum Dis 2022:annrheumdis-2021-221761. 10.1136/annrheumdis-2021-221761 35701153

[7] Rudwaleit M, van der Heijde D, Landewé R, et al. The assessment of Spondyloarthritis International society classification criteria for peripheral Spondyloarthritis and for Spondyloarthritis in general. Ann Rheum Dis 2011;70:25–31. 10.1136/ard.2010.133645 21109520

[8] Karmacharya P, Ogdie A, Eder L. Psoriatic arthritis and the association with Cardiometabolic disease: a narrative review. Ther Adv Musculoskelet Dis 2021;13:1759720X21998279. 10.1177/1759720X21998279 PMC7934027 33737966

[9] Derksen VFAM, Ajeganova S, Trouw LA, et al. Rheumatoid arthritis phenotype at presentation differs depending on the number of Autoantibodies present. Ann Rheum Dis 2017;76:716–20. 10.1136/annrheumdis-2016-209794 28283528

[10] Kallberg H, Padyukov L, Plenge RM, et al. Gene-gene and gene-environment interactions involving HLA-Drb1, Ptpn22, and smoking in two Subsets of rheumatoid arthritis. Am J Hum Genet 2007;80:867–75. 10.1086/516736 17436241 PMC1852748

[11] Huizinga TWJ, Amos CI, van der Helm-van Mil AHM, et al. Refining the complex rheumatoid arthritis phenotype based on specificity of the HLA-Drb1 shared EPITOPE for antibodies to Citrullinated proteins. Arthritis Rheum 2005;52:3433–8. 10.1002/art.21385 16255021

[12] Mahdi H, Fisher BA, Källberg H, et al. Specific interaction between genotype, smoking and Autoimmunity to Citrullinated alpha-Enolase in the etiology of rheumatoid arthritis. Nat Genet 2009;41:1319–24. 10.1038/ng.480 19898480

[13] Irigoyen P, Lee AT, Wener MH, et al. Regulation of anti-cyclic Citrullinated peptide antibodies in rheumatoid arthritis: contrasting effects of HLA-Dr3 and the shared EPITOPE Alleles. Arthritis Rheum 2005;52:3813–8. 10.1002/art.21419 16320316

[14] Verpoort KN, van Gaalen FA, van der Helm-van Mil AHM, et al. Association of HLA-Dr3 with anti-cyclic Citrullinated peptide antibody-negative rheumatoid arthritis. Arthritis Rheum 2005;52:3058–62. 10.1002/art.21302 16200610

[15] Sigurdsson S, Padyukov L, Kurreeman FAS, et al. Association of a haplotype in the promoter region of the interferon regulatory factor 5 gene with rheumatoid arthritis. Arthritis Rheum 2007;56:2202–10. 10.1002/art.22704 17599733

[16] Annunziato F, Romagnani C, Romagnani S. The 3 major types of innate and adaptive cell-mediated Effector immunity. J Allergy Clin Immunol 2015;135:626–35. 10.1016/j.jaci.2014.11.001 25528359

[17] Sharma V, Pope BJ, Santiago NV, et al. Decreased levels of Stat1 and interferon-gamma-induced Stat1 Phosphorylation in rheumatoid arthritis Cd4 and Cd8 T cells. ACR Open Rheumatol 2021;3:277–83. 10.1002/acr2.11244 33779079 PMC8063148

[18] Kato M. New insights into IFN-gamma in rheumatoid arthritis: role in the era of JAK inhibitors. Immunol Med 2020;43:72–8. 10.1080/25785826.2020.1751908 32338187

[19] Pickens SR, Volin MV, Mandelin AM, et al. IL-17 contributes to angiogenesis in rheumatoid arthritis. J Immunol 2010;184:3233–41. 10.4049/jimmunol.0903271 20173024 PMC2857761

[20] Laan M, Cui ZH, Hoshino H, et al. Neutrophil recruitment by human IL-17 via C-X-C Chemokine release in the Airways. J Immunol 1999;162:2347–52. 9973514

[21] Chabaud M, Fossiez F, Taupin J-L, et al. Enhancing effect of IL-17 on IL-1-induced IL-6 and leukemia inhibitory factor production by rheumatoid arthritis Synoviocytes and its regulation by Th2 Cytokines. J Immunol 1998;161:409–14. 9647250

[22] Kim K-W, Kim H-R, Kim B-M, et al. Th17 Cytokines regulate Osteoclastogenesis in rheumatoid arthritis. Am J Pathol 2015;185:3011–24. 10.1016/j.ajpath.2015.07.017 26362732

[23] Fossiez F, Djossou O, Chomarat P, et al. T cell Interleukin-17 induces Stromal cells to produce proinflammatory and hematopoietic Cytokines. J Exp Med 1996;183:2593–603. 10.1084/jem.183.6.2593 8676080 PMC2192621

[24] Zrioual S, Ecochard R, Tournadre A, et al. Genome-wide comparison between IL-17A- and IL-17F-induced effects in human rheumatoid arthritis Synoviocytes. J Immunol 2009;182:3112–20. 10.4049/jimmunol.0801967 19234208

[25] Zrioual S, Toh M-L, Tournadre A, et al. IL-17Ra and IL-17Rc receptors are essential for IL-17A-induced ELR+ CXC Chemokine expression in Synoviocytes and are Overexpressed in rheumatoid blood. J Immunol 2008;180:655–63. 10.4049/jimmunol.180.1.655 18097068

[26] Katz Y, Nadiv O, Beer Y. “Interleukin‐17 enhances tumor necrosis factor Α–induced synthesis of Interleukins 1, 6, and 8 in skin and Synovial fibroblasts: A possible role as a “Fine‐Tuning cytokine” in inflammation processes”. Arthritis Rheum 2001;44:2176–84. 10.1002/1529-0131(200109)44:9<2176::aid-art371>3.0.co;2-4 11592383

[27] Taams LS, Steel KJA, Srenathan U, et al. IL-17 in the Immunopathogenesis of Spondyloarthritis. Nat Rev Rheumatol 2018;14:453–66. 10.1038/s41584-018-0044-2 30006601

[28] van Tok MN, van Duivenvoorde LM, Kramer I, et al. Interleukin-17A inhibition diminishes inflammation and new bone formation in experimental Spondyloarthritis. Arthritis Rheumatol 2019;71:612–25. 10.1002/art.40770 30390386

[29] Steel KJA, Srenathan U, Ridley M, et al. Polyfunctional, proinflammatory, tissue-resident memory phenotype and function of Synovial Interleukin-17A+Cd8+ T cells in Psoriatic arthritis. Arthritis Rheumatol 2020;72:435–47. 10.1002/art.41156 31677365 PMC7065207

[30] Menon B, Gullick NJ, Walter GJ, et al. IL-17+Cd8+ T-cells are enriched in the joints of patients with Psoriatic arthritis and correlate with disease activity and joint damage progression. Arthritis Rheumatol 2014;66:1272–81. 10.1002/art.38376 24470327 PMC4158887

[31] Kissel T, Reijm S, Slot LM, et al. Antibodies and B cells recognising Citrullinated proteins display a broad cross-reactivity towards other post-Translational modifications. Ann Rheum Dis 2020;79:472–80. 10.1136/annrheumdis-2019-216499 32041746

[32] Monahan RC, van den Beukel MD, Borggreven NV, et al. Autoantibodies against specific post-Translationally modified proteins are present in patients with lupus and associate with major neuropsychiatric manifestations. RMD Open 2022;8:e002079. 10.1136/rmdopen-2021-002079 35450955 PMC9024229

[33] Ponchel F, van Delft MAM, Xie X, et al. Anti-Carbamylated protein antibodies: are they useful for the diagnosis of rheumatoid arthritis Clin Exp Rheumatol 2021;39:146–50. 10.55563/clinexprheumatol/u891rd 32662401

[34] Myasoedova E, Davis J, Matteson EL, et al. Is the epidemiology of rheumatoid arthritis changing? results from a population-based incidence study, 1985-2014. Ann Rheum Dis 2020;79:440–4. 10.1136/annrheumdis-2019-216694 32066556 PMC7085464

[35] Lee K, Min HK, Koh S-H, et al. Prognostic signature of interferon-gamma and Interleurkin-17A in early rheumatoid arthritis. Clin Exp Rheumatol 2022;40:999–1005. 10.55563/clinexprheumatol/mkbvch 34369364

[36] Cañete JD, Martínez SE, Farrés J, et al. Differential Th1/Th2 cytokine patterns in chronic arthritis: interferon Γ is highly expressed in Synovium of rheumatoid arthritis compared with Seronegative Spondyloarthropathies. Ann Rheum Dis 2000;59:263–8. 10.1136/ard.59.4.263 10733472 PMC1753106

[37] Partsch G, Wagner E, Leeb BF, et al. T cell derived Cytokines in Psoriatic arthritis Synovial fluids. Ann Rheum Dis 1998;57:691–3. 10.1136/ard.57.11.691 9924213 PMC1752505

[38] Moon J-S, Younis S, Ramadoss NS, et al. Cytotoxic Cd8^+^ T cells target Citrullinated antigens in rheumatoid arthritis. Nat Commun 2023;14:319. 10.1038/s41467-022-35264-8 36658110 PMC9852471

[39] Argyriou A, Wadsworth MH, Lendvai A, et al. Single cell sequencing identifies Clonally expanded Synovial Cd4(+) T(PH) cells expressing Gpr56 in rheumatoid arthritis. Nat Commun 2022;13. 10.1038/s41467-022-31519-6 PMC9279430 35831277

[40] Humby F, Lewis M, Ramamoorthi N, et al. Synovial cellular and molecular signatures stratify clinical response to csDMARD therapy and predict radiographic progression in early rheumatoid arthritis patients. Ann Rheum Dis 2019;78:761–72. 10.1136/annrheumdis-2018-214539 30878974 PMC6579551

[41] Wu X, Liu Y, Jin S, et al. Single-cell sequencing of immune cells from Anticitrullinated peptide antibody positive and negative rheumatoid arthritis. Nat Commun 2021;12. 10.1038/s41467-021-25246-7 PMC8371160 34404786

[42] Siebert S, McGucken A, McInnes IB. The IL-23/IL-17A axis in Spondyloarthritis: Therapeutics informing pathogenesis Curr Opin Rheumatol 2020;32:349–56. 10.1097/BOR.0000000000000719 32412997

[43] Taams LS. Interleukin-17 in rheumatoid arthritis: trials and tribulations. J Exp Med 2020;217:e20192048. 10.1084/jem.20192048 32023342 PMC7062523

[44] Kampylafka E, Simon D, d’Oliveira I, et al. Disease Interception with Interleukin-17 inhibition in high-risk psoriasis patients with Subclinical joint inflammation-data from the prospective IVEPSA study. Arthritis Res Ther 2019;21:178. 10.1186/s13075-019-1957-0 31349876 PMC6659205

[45] Schett G, Elewaut D, McInnes IB, et al. How cytokine networks fuel inflammation: toward a cytokine-based disease Taxonomy. Nat Med 2013;19:822–4. 10.1038/nm.3260 23836224

