# Supplementary material for: Type 17-specific immune pathways are active in early spondyloarthritis
Source: RMD Open. 2023 Dec 20;9(4):e003328. doi: 10.1136/rmdopen-2023-003328 (PMC10748989; doi:10.1136/rmdopen-2023-003328)
Supplement: Supplementary data [file rmdopen-2023-003328supp001.pdf]

Hughes, Ryan, Steel et al. Supplementary Table 1

No.	Original diagnosis at time of appt	Month + year of initial diagnosis	Final diagnosis	Month + year of consensus diagnosis by 2 independent clinicians
1	ReA	Feb-18	PsA	Sep-21 new clin symp
2	PsA	Jul-18	PsA	Sep-21
3	PsA	Jun-19	PsA	Sep-21
4	PsA	May-19	Undifferentiated IA	Sep-21 Rash not PsO
5	ReA	Aug-14	ReA	Sep-21
6	ReA	Jan-19	ReA	Sep-21
7	ReA	Aug-14	ReA	Sep-21
8	ReA	May-18	Undifferentiated IA	Sep-21 Inf Hx unclear
9	ReA	Jan-18	Undifferentiated IA	Sep-21 Inf Hx unclear
10	UIA	Dec-18	Undifferentiated IA	Sep-21
11	IA	Dec-15	Undifferentiated IA	Sep-21
12	UIA	Feb-14	Undifferentiated IA	Sep-21
13	EIA	Jun-19	ReA	Sep-21 Inf Hx clear
14	RA	Mar-19	Sero+ RA	Sep-21
15	RA	Mar-19	Sero+ RA	Sep-21
16	RA	May-19	Sero+ RA	Sep-21
17	RA	Nov-18	Sero+ RA	Sep-21
18	IA	Apr-16	Undifferentiated IA	Sep-21
19	Enteropathic SpA	Jul-19	Enteropathic SpA	Sep-21
20	RA	Nov-20	Sero+ RA	Sep-21
21	EIA	Oct-18	Undifferentiated IA	Sep-21
22	PsA	Nov-19	PsA	Sep-21
23	PsA	Nov-20	PsA	Sep-21
24	PsA	May-21	PsA	Sep-21
25	IA	Feb-15	PsA	Sep-21 new clin symp
26	ReA	Jan-20	ReA	Sep-21
27	ReA	Aug-18	Undifferentiated IA	Sep-21 Inf Hx unclear
28	ReA	Mar-19	Undifferentiated IA	Sep-21 Inf Hx unclear
29	Sero+ RA	Jul-19	Sero+ RA	Sep-21
30	Enteropathic SpA	Nov-19	Enteropathic SpA	Sep-21

Supplementary Table 1. Original and final diagnosis and their dates for all of the EIA patients included in the study.
Abbreviations: IA, inflammatory arthritis; Inf Hx, infection history; PsA, psoriatic arthritis; PsO, psoriasis; RA, rheumatoid arthritis; ReA, reactive arthritis; SpA, spondyloarthritis.

Hughes, Ryan, Steel et al. Supplementary Figure 1

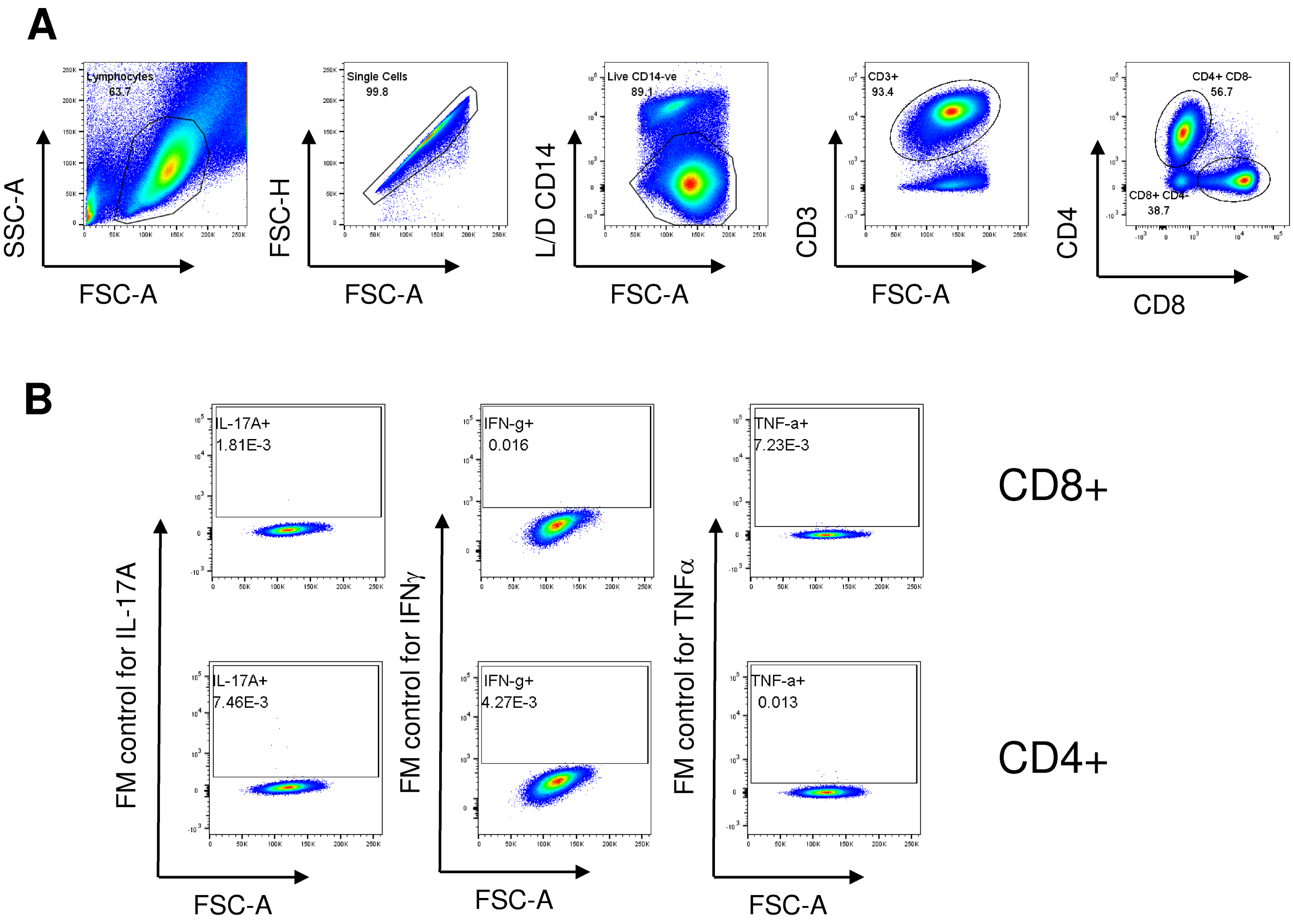

Supplementary Figure 1. Gating strategy for analysis of cytokine-expressing CD8+ and CD4+ T cells. (A) SFMC were identified based on their FSC and SSC profile. This was followed by single cell gating and dead cell and CD14+ cell exclusion. CD3+ T cells were then gated, followed by CD8+CD4- and CD4+CD8- T cell identification. (**B**) For FM control staining, small sample aliquots were combined. Cells were stained as described in the Methods but without any anti-cytokine mAbs. FM gates were then set to aid the determination of cytokine-expressing T cells.

Hughes, Ryan, Steel et al. Supplementary Figure 2

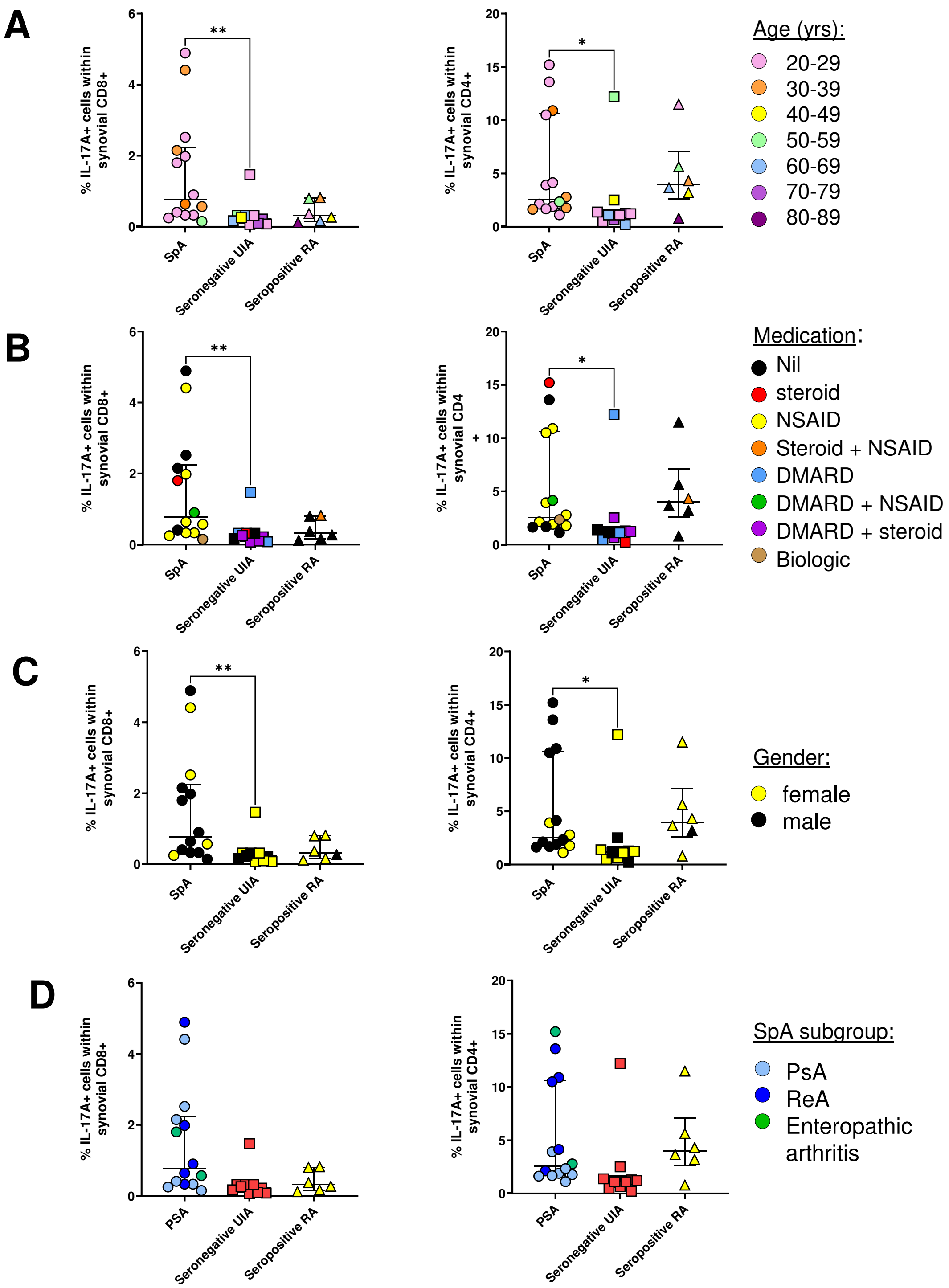

Supplementary Figure 2. IL-17A+ cells within synovial CD8+ (left) or CD4+ (right) T cells coloured according to demographic or clinical data. (A) Coloured according to age: 20-29 (pink), 30-39 (orange), 40-49 (yellow), 50-59 (green), 60-69 (blue), 70-79 (purple), 80-89 (dark purple). (**B**) Coloured according to treatment: no treatment (black), steroid (red), NSAID (yellow), steroid + NSAID (orange), DMARD (blue), DMARD + NSAID (green), DMARD + steroid (purple), biologic (brown). (**C**) Coloured according to gender: female (pink), male (blue). (**D**) Coloured according to SpA subgroup: PsA (light blue), reactive arthritis (dark blue), enteropathic arthritis (green).

Hughes, Ryan, Steel et al. Supplementary Figure 3

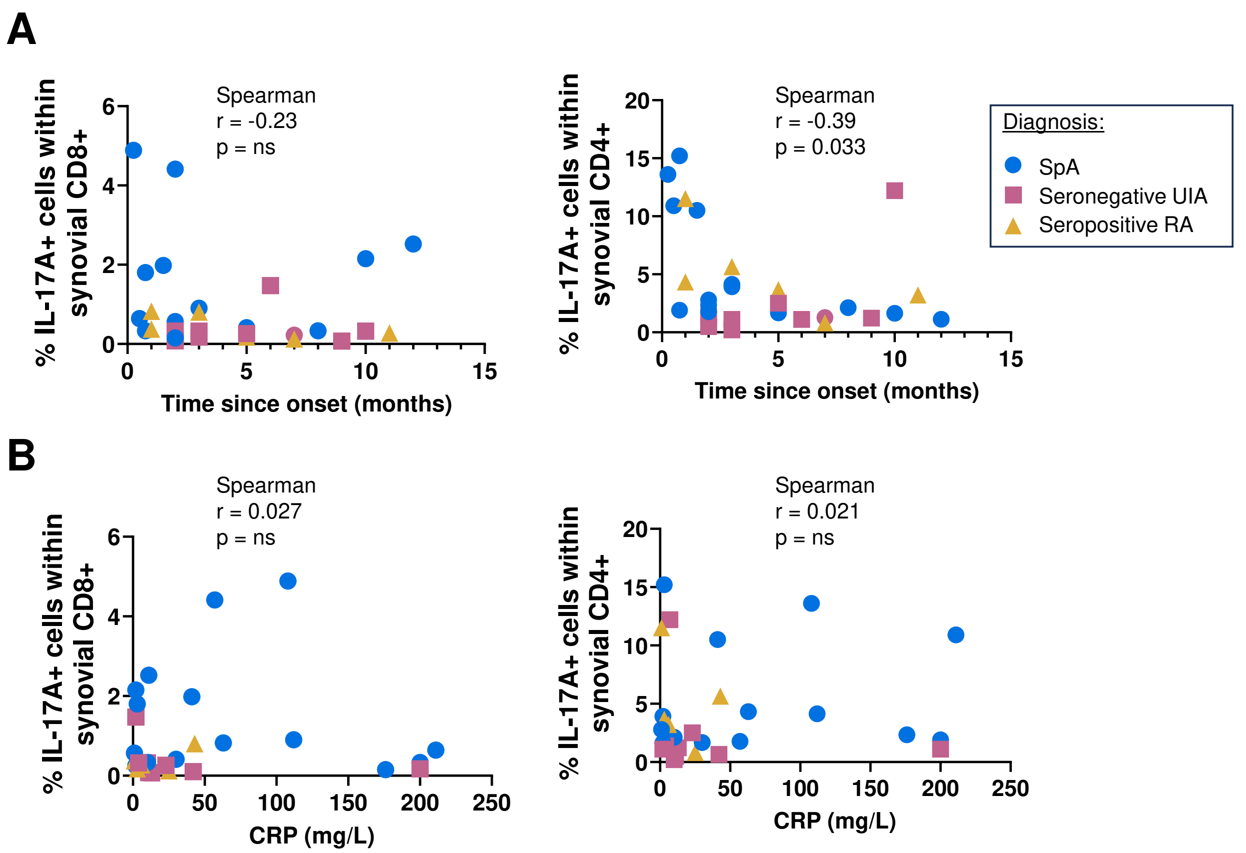

Supplementary Figure 3. Correlations between IL-17A+ cells in synovial CD8+ or CD4+ T cells and clinical data. IL-17A+ cells within synovial CD8+ (left) or CD4+ (right) T cells plotted against time since onset of first symptoms (A, n=30) and against CRP values (B, n=30), lower limit for CRP detection = 1mg/L.

Hughes, Ryan, Steel et al. Supplementary Figure 4

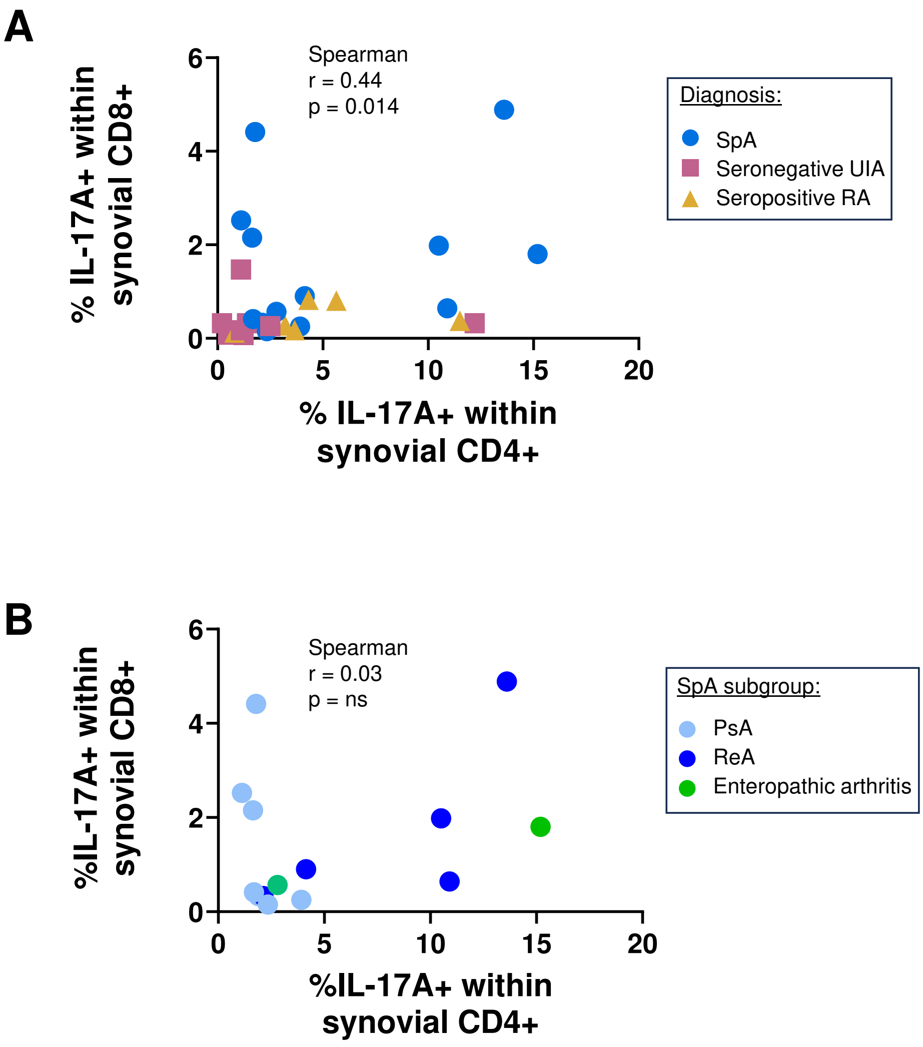

Supplementary Figure 4. Correlations between IL-17A+ cells in synovial CD8+ and CD4+ T cells. Correlation between IL-17A+ cells within synovial CD8+ and CD4+ T cells, for all EIA samples analysed (A, n=30) or in SpA subtypes only (B, n=14).
